# Forward genetics for back-in-time questions

**DOI:** 10.7554/eLife.05218

**Published:** 2014-11-04

**Authors:** Alex de Mendoza, Iñaki Ruiz-Trillo

**Affiliations:** 1 **Alex de Mendoza** is in the Institut de Biologia Evolutiva (CSIC-Universitat Pompeu Fabra), Barcelona, Spain; Departament de Genètica, Universitat de Barcelona, Barcelona, Spainalex.demendoza@ibe.upf-csic.es; 2 **Iñaki Ruiz Trillo** is in the Institut de Biologia Evolutiva (CSIC-Universitat Pompeu Fabra), Barcelona, Spain; Departament de Genètica, Universitat de Barcelona, Barcelona, Spain; Institució Catalana de Recerca i Estudis Avançats (ICREA), Barcelona, Spaininaki.ruiz@ibe.upf-csic.es

**Keywords:** choanoflagellate, Salpingoeca rosetta, multicellularity, C-type lectin-like, rosetteless, other

## Abstract

A genetic screen has revealed one of the molecules that allow choanoflagellates, the closest unicellular relative of animals, to form colonies, which could help researchers to answer questions about the earliest days of animal evolution.

**Related research article** Levin TC, Greaney AJ, Wetzel L, King N. 2014. The *rosetteless* gene controls development in the choanoflagellate *S. rosetta*. *eLife* **3**:e04070. doi: 10.7554/eLife.04070

**Image** Choanoflagellates need a protein (cyan) encoded by the *rosetteless* gene to form colonies

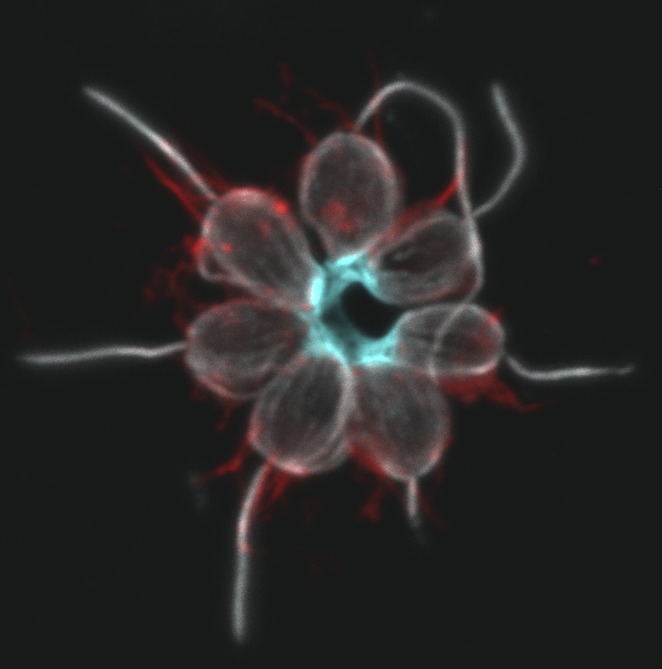

The central question in the field of developmental biology is: how can a single fertilised egg cell develop into a living organism made of millions of specialised cells? The evolutionary flip side of this question is: how did a multicellular animal first evolve from a single-celled microorganism? For decades evolutionary biologists have tried to answer this question by analysing the fossil record, by studying the species that represent the earliest branches in the animal family tree and, lately, by exploring the closest unicellular relatives of the animal kingdom.

Choanoflagellates are considered to be the closest single-celled cousins of animals that are alive today. Choanoflagellates are unicellular eukaryotes that live in fresh and marine waters, and each beats its tail-like flagellum to catch bacterial prey. The choanoflagellates' close relationship with the animals was first hypothesised as early as two centuries ago, and was based on their similarity to a specific cell type found in one of the simplest animals, the sponges (Nielsen, 2008). Interestingly, some species of choanoflagellates form colonies of several cells, and it has been suggested that these colonies resemble the putative ancestor of all animals (Richter and King, 2013). And although it is far from clear that the common ancestor of choanoflagellates and animals had the ability to form colonies, understanding how these unicellular cousins regulate their own development might provide insights into the first steps of multicellularity.

The interest in choanoflagellate development has grown in recent years, thanks largely to data provided by the analysis of the genome sequences of two choanoflagellates, *Monosiga brevicollis* and *Salpingoeca rosetta* (King et al., 2008; Fairclough et al., 2013). These genomes contain genes for proteins that are important for animal development and cell signalling; these proteins include the cadherins, which are involved in cell-cell adhesion (Abedin and King, 2008). The presence of these two features—genes related to multicellularity and a colonial stage—in choanoflagellates raised an intriguing question: do animals and choanoflagellates use the same genes to become multicellular? If the answer to this question is yes, choanoflagellate development may yet reveal ancestral mechanisms that paved the way for the origin of animals.

Nevertheless, a persistent problem that has hindered attempts to address this question has been the limited number of genetic tools available to study the functions of genes in choanoflagellates. Studies based on transcriptomics and immunohistochemistry techniques had offered insights regarding the regulation of gene expression and the localisation of proteins but, unfortunately, such analyses failed to provide a clear link between gene function and colonial development. Now in *eLife*, Nicole King and colleagues at the University of California, Berkeley—including Tera Levin as the first author—report that they have solved this problem by performing a genetic screen in *S. rosetta* (Levin et al., 2014). This screen has provided the first evidence that links a genetic change to an observable defect in *S. rosetta*'s development: in other words, it has linked genotype to phenotype in a choanoflagellate that forms colonies.

Levin et al. used the classic approach of creating random mutations throughout the *S. rosetta* genome, and then isolating mutant lines that were not able to form colonies (Figure 1). They could reliably select for this phenotype because *S. rosetta* can be made to form rosette-like colonies in the laboratory by adding a molecule, called a sulfunolipid, which is produced by a bacterial species (Alegado et al., 2012). After screening thousands of mutants, Levin et al. isolated nine that did not form colonies in the presence of the bacteria, but that otherwise appeared identical to the wild-type cells.

**Figure 1.**
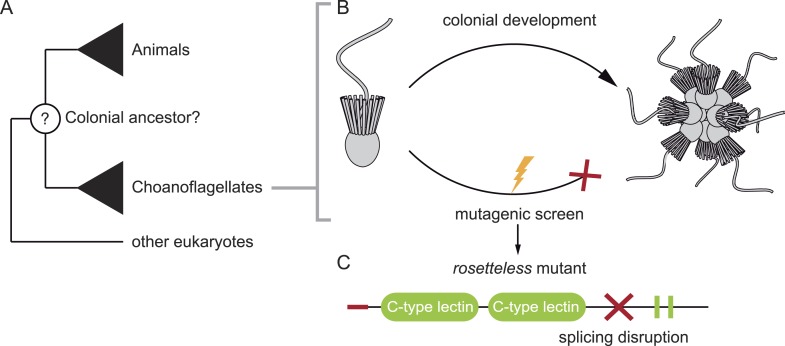
Studying our unicellular cousins to understand the early ancestor of all animals. (**A**) Choanoflagellates are one of the closest living relatives of animals. (**B**) The choanoflagellate *Salpingoeca rosetta* forms rosette-like colonies, and Levin et al. have performed a random mutagenesis screen on *S. rosetta* (lightening bolt) and selected for strains that were unable to develop colonies. (**C**) One of the mutants identified (which Levin et al. named *rosetteless*) was affected in a protein with a signal peptide (red tip), ‘C-type lectin’ domains (green ovals) and two internal repeats (green rectangles). The red cross indicates where the mutation in the gene disrupts the normal processing (i.e. splicing) of the mRNA transcript. This introduces a premature stop into the transcript, which typically results in truncated versions of the protein being produced.

FIGURE CREDIT: 3D illustrations by Mark Dayel (CC BY-SA 3.0) www.dayel.com/blog/2010/10/07/choanoflagellate-illustrations.

Levin et al. then focused on one specific mutant strain, which they named ‘*rosetteless*’. To identify the mutation responsible for the ‘*rosetteless*’ phenotype they sequenced the mutant’s genome and compared it to the genome of the wild-type. Few nucleotides were different, and when Levin et al. crossbred this mutant with a wild-type strain, by the second generation they had identified the specific mutation linked to the trait. The affected gene codes for a type of protein known as a C-type lectin, with the mutation causing truncated versions of the protein to be produced (Figure 1).

To validate the connection between the C-type lectin and the developmental phenotype, Levin et al. raised an antibody that binds to, and blocks, the protein and showed that wild-type cells could not form colonies when they were cultured with this antibody. Moreover, the C-type lectin, encoded by the ‘*rosetteless*’ gene, localised at the core of the colonies.

C-type lectins are found in many eukaryotes and are very abundant in animal genomes. Interestingly, animal C-type lectins are involved in cell-cell adhesion and the immune system (Geijtenbeek and Gringhuis, 2009). Thus, it is tempting to assume that the ancestor of both animals and choanoflagellates had a version of the *rosetteless*-encoded protein. However, the evolutionary history of this protein is difficult to trace, and conserved copies of this protein have not been identified in the genomes of animals or other choanoflagellates. Therefore it remains unclear whether the *rosetteless* gene is related to those that encode animal C-type lectins or whether both animals and choanoflagellates have independently evolved to use similar proteins with distinct origins to perform similar functions.

Regardless of whether the *rosettless* gene and genes for animal C-type lectins have evolved from a common ancestor, or have converged on a similar solution to two similar problems Levin et al.'s study opens the door to new avenues of research on choanoflagellate biology. Characterising the other mutants identified in the screen will surely reveal more developmental genes in choanoflagellates. Similar studies of other unicellular cousins of animals are also likely to provide crucial insights: for example, proteins called integrins (which are also found in animals) are known to be involved in cell-cell adhesion in the multicellular aggregative stage of the amoeba *Capsaspora owczarzaki* (Sebé-Pedrós et al., 2013).

Finding common tools involved in the multicellular life cycles of animals and their unicellular cousins is key to unravelling how complex the last common single-celled ancestor of animals was. Knowing this will allow us to answer the decisive question: was the origin of multicellular animals one giant leap in eukaryotic evolution or one small step for a colony-forming microorganism?
